# Impact of infrared moxibustion for patients with mild hyperlipidemia: a protocol for a randomized controlled trial

**DOI:** 10.1080/07853890.2026.2665513

**Published:** 2026-05-04

**Authors:** Haihua Xie, Fang Feng, Jiaxing Zhu, Wei Zhu, Shulin Xiong, Bowen Xing, Sihao Chen, Qingxia Li, Ting Zhu, Mi Liu

**Affiliations:** a College of Acupuncture & Tuina and Rehabilitation, Hunan University of Chinese Medicine, Changsha, China; b Preventive Treatment of Disease Center, Chenzhou First People’s Hospital, Chenzhou, China; c Department of Acupuncture and Tuina, Hunan Integrated Traditional Chinese and Western Medicine Hospital, Changsha, China; d Preventive Treatment of Disease Center, The Second Affiliated Hospital of Hunan University of Chinese Medicine, Changsha, China; e College of Acupuncture & Tuina and Rehabilitation, The Second Affiliated Hospital of Hunan University of Chinese Medicine, Changsha, China

**Keywords:** Infrared moxibustion, hyperlipidemia, microbiota-gut-brain axis

## Abstract

**Introduction:**

Hyperlipidemia serves as a significant risk factor for cardiovascular diseases and cognitive impairment, making early intervention and management highly necessary. Infrared moxibustion, a modern complementary and alternative therapy, currently lacks high-quality clinical research to substantiate its efficacy and underlying mechanisms. Therefore, this study aims to evaluate the effects of infrared moxibustion on blood lipid levels in patients with hyperlipidemia, and to explore its potential mechanisms by analyzing changes in gut microbiota and their metabolites, as well as alterations in brain gray matter volume.

**Patient and methods:**

This study will recruit 158 patients with mild hyperlipidemia and randomly assign them to either a genuine infrared moxibustion group or a sham infrared moxibustion group. The intervention will be administered twice weekly for a total of 8 weeks. Blood lipid levels, Montreal Cognitive Assessment scores, Hamilton Depression Rating Scale scores, Hamilton Anxiety Scale scores, and Pittsburgh Sleep Quality Index scores will be compared between the two groups before treatment, and at 4, 8, and 12 weeks post-treatment. Additionally, changes in gut microbiota, the gut microbiota metabolite trimethylamine N-oxide (TMAO), and brain gray matter volume will be detected and compared between the two groups before treatment and at 8 weeks post-treatment (See Graphic Abstract).

## Graphical Abstract

**Figure:**
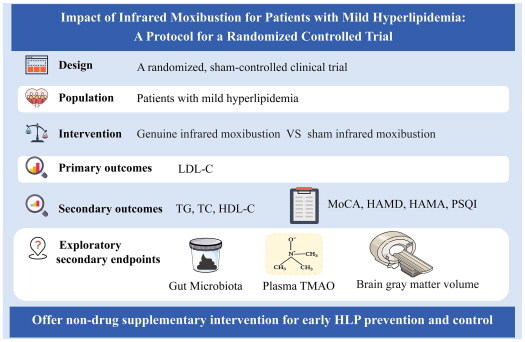

## 1. Introduction

Hyperlipidemia (HLP) refers to a systemic lipid metabolism abnormality characterized by elevated levels of total cholesterol (TC), triglycerides (TG), and/or low-density lipoprotein cholesterol (LDL-C), and/or reduced levels of high-density lipoprotein cholesterol (HDL-C) in the plasma due to various causes. HLP, particularly elevated LDL-C levels, has been established as a risk factor for cardiovascular diseases and cognitive disorders (CD) [1,2]. While its role in the former has been extensively studied, its contribution to the latter remains to be explored. It has been reported that alterations in plasma lipid profiles appear to exacerbate cognitive decline, thereby increasing the risk of developing Alzheimer’s disease in non-demented elderly individuals [3–6]. This could be attributed to the fact that dyslipidemia negatively impacts hippocampal synaptic plasticity by promoting the accumulation of Aβ and TRPM2 [7,8], activating microglia, exerts a detrimental impact on hippocampal synaptic plasticity [9], facilitates hippocampal atrophy [10–12], mediates large-scale brain network abnormalities [13], and ultimately intensifies cognitive decline. Cognitive decline weakens individuals’ abilities in memory, thinking, comprehension, coordination, communication, and more. Early intervention targeting HLP may reduce the risk of developing CD [14]. Therefore, in-depth research into the underlying pathogenesis and effective therapeutic approaches for HLP holds crucial significance for improving the prevention and treatment of CD.

Currently, statins serve as the mainstream drugs for clinical treatment of hyperlipidemia, with proven lipid-lowering efficacy. Although some early studies have reported a potential association between statins and cognitive decline [15], current evidence-based research generally indicates that these drugs have a neutral effect on cognition and may even confer a protective effect. However, in clinical practice, some patients experience varying degrees of drug intolerance, such as muscle side effects [16,17], and low medication adherence [18]. Furthermore, despite the active recommendation of lifestyle interventions by clinical guidelines, the actual implementation and persistence among patients in real-world clinical settings remain unsatisfactory. Therefore, this highlights the urgent clinical need to explore effective and safe complementary or alternative therapies.

Notably, moxibustion is utilized in clinical practice as an adjunctive treatment for HLP. Preliminary clinical studies and systematic reviews suggest that moxibustion may offer potential benefits in regulating blood lipids [19–21]. However, the overall quality of existing research is generally suboptimal, with issues such as small sample sizes, inconsistent methodological quality and the absence of a double-blind design. Furthermore, traditional moxibustion is fraught with limitations, including smoke irritation from moxa and challenges in precisely controlling temperature. In contrast, infrared moxibustion (IM), a modern and innovative moxibustion technique, integrates the dual therapeutic effects of infrared radiation and moxibustion without the associated smoke irritation. Nevertheless, the current evidence supporting the use of IM for the treatment of hyperlipidemia is relatively weak, and its therapeutic mechanisms remain to be further explored.

An increasing number of scholars have proposed correlations between gut microbiota and their metabolites with HLP. Dysbiosis of the gut microbiota may serve as a mediating factor in the causal relationship between a high-calorie diet and CD [22]. Xia M et al. revealed that, compared to healthy volunteers, the mixed fecal samples from elderly HLP patients were predominantly composed of *Firmicutes* and *Proteobacteria*, with an increased relative abundance of *Helicobacter, Lactobacillus*, and *Desulfovibrio*, and a decreased relative abundance of *Parabacteroides, Bacteroides, Blautia, Bifidobacterium*, and *Peptococcus* [23]. Among the metabolites produced by the gut microbiota, trimethylamine-N-oxide (TMAO) has garnered considerable attention for its involvement in the regulation of lipid metabolism and inflammatory responses, and is recognized as a risk factor for HLP [24]. Numerous animal experiments and clinical studies have demonstrated that a high-fat diet increases TMAO production [25–27]. Fei’erdun T et al. discovered that HLP patients exhibited higher plasma levels of TMAO compared to those with normal lipid profiles, with its concentration showing a positive correlation with TG and a negative correlation with high-density lipoprotein cholesterol HDL-C [27]. A high-fat diet may trigger or exacerbate the onset of cognitive impairment by increasing the abundance of harmful bacterial genera and TMAO levels, while simultaneously reducing the abundance of beneficial bacterial genera [28]. Our previous research indicated that TMAO is involved in the pathogenesis of mild cognitive impairment (MCI) and can serve as a biomarker and therapeutic target for MCI [29]. Furthermore, TMAO and its precursor substances have been confirmed by multiple studies to be associated with brain volume changes, cognitive decline, and an increased risk of dementia [30–32]. Yaqub A and colleagues proposed that elevated levels of TMAO-related metabolites may induce brain atrophy, thereby increasing the risk of dementia [30]. Existing evidence shows that traditional moxibustion can reduce the levels of TMAO in animals fed a high-fat diet and improve their lipid profiles. Moreover, changes in its precursor substances exhibit a significant correlation with specific bacterial groups [33,34]. Moreover, a study has observed that acupuncture intervention can induce alterations in brain structure in obese patients and animals on a high-fat diet [35], suggesting that acupuncture and moxibustion therapies may participate in metabolic regulation by influencing the central nervous system.

Therefore, we designed this randomized, double-blind clinical study. We hypothesize that infrared moxibustion may decrease blood lipid levels in patients with mild hyperlipidemia. The underlying mechanisms may be associated with the modulation of intestinal microbiota abundance, the reduction of TMAO levels, and the mitigation of cerebral atrophy. This study holds significant importance in reducing the risk of CD in HLP patients, serving as an early preventive measure. Furthermore, the findings of this study can offer a novel non-pharmacological therapeutic option for the clinical management of HLP, possessing substantial scientific value and broad clinical application prospects.

## 2. Patients and methods

### 2.1. Study design

**Figure 1.**
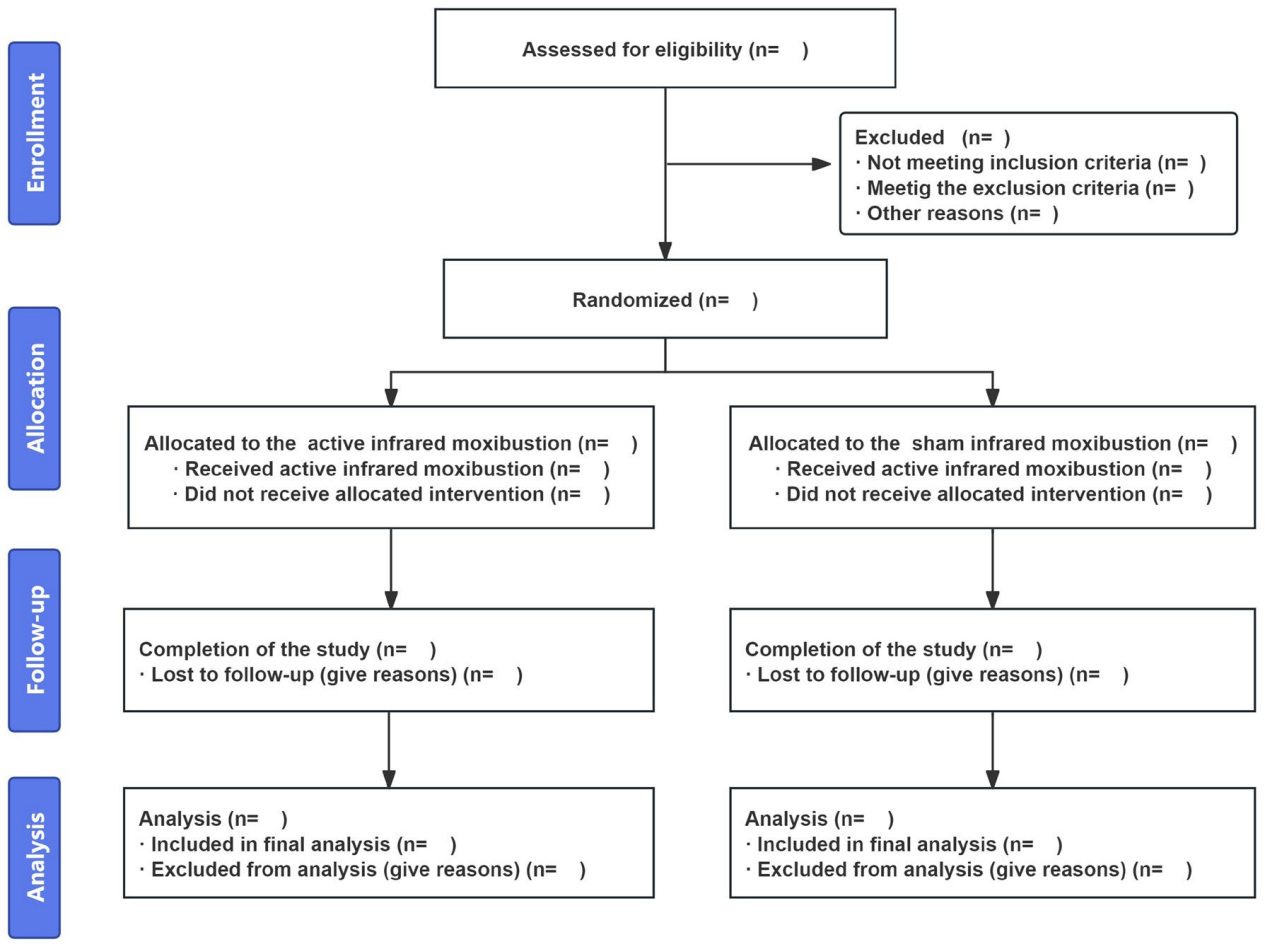
Flow diagram.

This study will conduct a prospective, multicenter, double-blind randomized controlled trial at the Second Affiliated Hospital of Hunan University of Chinese Medicine, Hunan Integrated Traditional Chinese and Western Medicine Hospital, and Chenzhou First People’s Hospital. The study has received approval from the Ethics Committee of the Second Affiliated Hospital of Hunan University of Chinese Medicine (Approval Certificate Numbers: KY-2025-023-01, [2025] No. 371, (Research) No. 2025100), and has been registered on the International Traditional Medicine Clinical Trial Registry Platform (https://itmctr.ccebtcm.org.cn/mgt/search; Registration Number: ITMCTR2025001463). This study will be reported in accordance with the Declaration of Helsinki and the Standard Protocol Items: Recommendations for Interventional Trials (SPIRIT) guidelines. We plan to recruit 158 eligible participants from August 2025 to June 2027. After signing informed consent forms, they will be randomly assigned to two groups: the infrared moxibustion group (IM group) and the sham infrared moxibustion group (Sham IM group), as detailed in Figure 1.

### 2.2. Randomization, allocation concealment, and blinding

Central stratified randomization will be employed using SAS 9.3 (SAS Institute Inc., Cary, NC, USA) statistical software to generate random numbers. Eligible participants will be randomly assigned to the IM group and the sham IM group in a 1:1 ratio through a central randomization system. The central randomization system will be managed by independent personnel who will not be involved in participant recruitment, intervention delivery, outcome assessment, or statistical analysis to prevent selection bias. Both participants and the treating physicians will be blinded to the treatment allocation between active infrared moxibustion and sham infrared moxibustion. Throughout the data collection and analysis process, assessors and statisticians will also be blinded to the treatment allocation.

### 2.3. Participants

#### 2.3.1. Diagnosis criteria

Participants must meet the diagnostic criteria for mild hyperlipidemia [36]. Mild hyperlipidemia can be diagnosed if patients meet one or more of the following fasting venous plasma test indicators: (1) 2.3 mmol/L > TG ≥ 1.7 mmol/L; (2) 6.2 mmol/L > TC ≥ 5.2 mmol/L; (3) 4.1 mmol/L > LDL-*C* ≥ 3.4 mmol/L.

#### 2.3.2. Inclusion criteria

(1) Meet the diagnostic criteria for hyperlipidemia; (2) Aged between 18 and 70 years; (3) Not currently using lipid-lowering medications, or have discontinued lipid-lowering medications for at least one month, and are not participating in other clinical studies; (4) Sign an informed consent form.

#### 2.3.3. Exclusion criteria

(1) Secondary hyperlipidemia caused by other diseases (e.g. nephrotic syndrome, gout, hypothyroidism, acute or chronic hepatobiliary diseases, diabetes); (2) Concomitant severe primary diseases of the liver, heart, or brain; (3) Allergic to infrared therapy; (4) Have undergone gallbladder surgery or major gastrointestinal surgery within the past six months; (5) Pregnant or lactating women; (6) Poor compliance.

### 2.4. Interventions

#### 2.4.1. IM group

Participants in the observation group will receive infrared moxibustion intervention using an infrared moxibustion chamber (Model CH-6, Hunan Cihui Medical Technology Co., Ltd., Changsha, Hunan). Participants will lie supine with their entire body below the head and neck placed inside the infrared chamber. Infrared moxibustion will be initiated with a single button press. The temperature inside the chamber will be set to 50 °C, with a treatment duration of 40 min, once per day, twice per week, for a total of 8 weeks.

#### 2.4.2. Sham IM group

Participants in the control group will receive sham far-infrared moxibustion intervention. The treatment protocol will be identical to that of the treatment group, except that the device will not emit infrared rays when turned on.

In addition, both groups of participants will receive therapeutic lifestyle changes, including a balanced diet, moderate increase in physical activity, weight control, smoking cessation, and limited alcohol consumption.

### 2.5. Outcomes

Professional assessors will observe primary and secondary outcomes in participants at four time points: baseline (T1), after 4 weeks of treatment (T2), after 8 weeks of treatment (T3), and 4 weeks after the end of treatment (T4) (Table 1). To ensure the quality of scale assessments and minimize inter-rater variability, assessors will undergo unified standardized training based on a pre-established operation manual before the study begins. Only after passing the training will, they be allowed to perform assessments. Each scale assessor will be responsible for only part of the assessment tasks and will be unaware of the participants’ group assignments. They will also not be involved in the intervention process or the final data statistical analysis.

**Table 1.** Trial implementation schedule.

	Study period					
	Enrollment	Allocation	Treatment phase			Follow-up
Timepoint	−1 week		0 day	4 week	8 week	12 week
**Enrollment**						
Eligibility screen	X					
Informed consent	X					
Allocation		X				
**Interventions**						
Active infrared moxibustion			X	X	X	
Sham infrared moxibustion			X	X	X	
**Assessments**						
Blood lipids		X		X	X	X
MoCA		X		X	X	X
HAMD		X		X	X	X
HAMA		X		X	X	X
PSQI		X		X	X	X
Adverse events			X	X	X	
Gut Microbiota		X			X	
Plasma TMAO		X			X	
MRI Acquisition		X			X	

MoCA: Montreal Cognitive Assessment; HAMD: Hamilton Depression Rating Scale; HAMA: Hamilton Anxiety Scale; PSQI: Pittsburgh Sleep Quality Index; TMAO: trimethylamine-N-oxide; MRI: Magnetic resonance imaging.

#### 2.5.1. Primary outcomes

The change in LDL-C from baseline to 8 weeks after intervention.

#### 2.5.2. Secondary outcomes

1. Other lipid profile indicators: Triglycerides, total cholesterol, and high-density lipoprotein cholesterol.
2. Montreal Cognitive Assessment (MoCA): Suitable for rapid screening of mild cognitive impairment and other cognitive declines, with higher scores indicating better cognitive function.
3. Hamilton Depression Rating Scale (HAMD): Commonly used to assess clinical depressive symptoms. The scale consists of 17 items with a total score of 54. Scores of 7–17 indicate mild depression, 18–24 indicate moderate depression, and >25 indicate severe depression. Higher scores indicate more severe depression.
4. Hamilton Anxiety Scale (HAMA): Commonly used to assess clinical anxiety symptoms. The scale consists of 14 items with a total score of 70. Higher scores indicate more severe anxiety.
5. Pittsburgh Sleep Quality Index (PSQI): Used to assess sleep quality. The scale consists of 24 items, with higher scores indicating poorer sleep quality.
6. Safety: All adverse events occurring during the research process will be recorded by experienced acupuncturists, including their occurrence time, severity, and the measures taken for management. Potential study-related adverse events mainly encompass skin scalds and discomforts that may arise after moxibustion, such as palpitations, dizziness, etc. Researchers, in collaboration with acupuncturists, will analyze whether the occurrence of adverse events is related to the study intervention.

#### 2.5.3. Exploratory secondary endpoints

##### 2.5.3.1. Gut microbiota

Fecal samples will be collected from participants at baseline and after 8 weeks of treatment and stored in a −80 °C freezer. Total genomic DNA of the fecal microbiota will be extracted according to the QIAamp Fast DNA Stool Mini Kit operation manual. DNA concentration and purity will be assessed using a microplate reader, and DNA integrity will be detected by gel electrophoresis. The 16S V3-V4 region will be amplified using 341 F and 806 R primers, and 16S library construction will be performed according to the Illumina library construction strategy. Preliminary quantification will be performed using Qubit 3.0, library insert size will be detected using Agilent 2100, and accurate quantification will be performed using Q-PCR. After the library passes quality control, MiniSeq high-throughput sequencing will be performed. Alpha diversity will be assessed using the Observed species, Chao1, Shannon, and Simpson indices. Beta diversity will be compared using weighted and unweighted UniFrac distances, and differences in microbiota structure within and between groups will be evaluated using principal coordinate analysis (PCoA) in R software (version 3.5.3).

##### 2.5.3.2. Plasma TMAO

Participants will fast starting at 8 PM the night before blood collection. At 8 AM the next morning, a 6 mL fasting venous blood sample will be drawn from the elbow vein after 12 h of fasting. Of this, 3 mL will be centrifuged to obtain serum for lipid measurement; the other 3 mL will be anticoagulated and centrifuged to obtain plasma samples, which will be immediately placed in a −80 °C freezer for TMAO detection. Changes in TMAO levels in participants will be detected at baseline and after 8 weeks of treatment. Plasma TMAO content will be detected using liquid chromatography-mass spectrometry, with TMAO content quantified by peak area. Larger peak areas indicate higher TMAO content.

##### 2.5.3.3. MRI acquisition and analysis

MRI scans were performed at baseline and 8 weeks after the intervention. Magnetic resonance imaging data will be acquired using a Siemens Verio 3.0 T MR scanner (Siemens, Germany). During scanning, participants will lie supine on the examination table with their head fixed and use specialized sponge ball earplugs to reduce noise. They will be instructed to breathe calmly, relax both physically and mentally, place their hands by their sides, and naturally separate their legs. They should maintain a fixed body position and avoid movement throughout the scanning process. Scanning parameters include: TR = 2530 ms, TE = 2.01 ms, flip angle (FA) = 7°, field of view (FOV) = 256 mm × 256 mm, matrix = 256 × 256, slice thickness = 1 mm.

Further processing of the T1 structural image data was performed using SPM12 software (Statistical Parametric Mapping, Wellcome Trust Centre for Neuroimaging, London, UK) and its CAT12 toolbox (Computational Anatomy Toolbox) on the MATLAB platform (The MathWorks Inc., Natick, MA, USA). The main steps included: (1) Data format conversion and preparation; (2) Segmentation of the images into gray matter, white matter, and cerebrospinal fluid; (3) Spatial registration and normalization to the Montreal Neurological Institute standard space using the DARTEL algorithm; (4) Image quality inspection; (5) Spatial smoothing with an 8 mm full-width at half-maximum Gaussian kernel.

### 2.6. Data collection and management

After full communication, participants will sign informed consent forms. Case report forms must be promptly completed and signed by assessors and corresponding researchers. After the project manager reviews the completed case report forms, data entry will be performed by two data entry clerks to reduce bias caused by data entry errors. The original case report forms will then be uniformly managed by the project manager.

### 2.7. Sample calculation

The sample size for this study was calculated using G*Power 3.1.9.7 software based on existing research on moxibustion for treating hyperlipidemia. According to previous trial results [37], the LDL-C levels after intervention in the moxibustion group and the control group were 4.41 ± 0.52 mmol/L and 3.72 ± 0.43 mmol/L, respectively. With a two-tailed test value of α = 0.05 and a power of 0.95, the sample size per group was calculated to be *n* = 66. Considering a 20% dropout rate, the required sample size per group is 79, resulting in a total required sample size of 158 for this study.

### 2.8. Statistics

All data obtained in this study will be analyzed using SPSS 25.0 (SPSS Inc., Chicago, IL, USA). The analysis will be conducted using the Intention-to-Treat (ITT) analysis approach. Continuous data that conform to a normal distribution will be described using the mean ± standard deviation; otherwise, the median (interquartile range) [Median (Inter Quartile Range), M(IQR)] will be used. Categorical data will be analyzed and described using frequencies. The specific analysis is as follows.

#### 2.8.1. Baseline data

To ensure comparability between the two groups prior to the intervention, we will compare differences in each outcome between the two groups at baseline. The t-test, non-parametric test, or chi-square test will be employed to compare the general demographic data, primary and secondary outcomes, and plasma TMAO levels between the two groups. Data will be analyzed using two-sided tests, with a significance level of α = 0.05. A *p* value of <0.05 indicates a statistically significant difference.

#### 2.8.2. Clinical observation outcomes

Missing values will be imputed using multiple imputation methods. Linear mixed models will be used to analyze data from the four measurement time points to compare group effects, time effects, and interaction effects on outcome indicators between the two groups. The model will include group, time, and the group × time interaction as fixed effects, with the subject set as a random intercept to account for the inherent correlation in repeated-measure data. The model will include variables that were unbalanced between groups at baseline as covariates to correct for the impact of baseline differences on treatment effect estimates and ensure the robustness of the results. If a significant interaction is detected, the Bonferroni corrections post-hoc test will be employed to further determine its significance, with the significance level set at *p* < 0.05 for a two-tailed test.

Given that the inclusion criteria of this study do not restrict cognition and emotional state, the study may be insufficiently sensitive to detecting improvements induced by the intervention. Based on this, we will conduct subgroup analyses focusing on subgroups with mild cognitive impairment (MoCA score < 26), depressive symptoms (HAMD score > 7), and anxious symptoms (HAMA score > 7), respectively. We will analyze the impact of the intervention on cognitive and emotional scores separately within these subgroups to further identify the populations that benefit from the intervention and to more comprehensively evaluate the potential benefits of the intervention.

#### 2.8.3. MRI analysis

1. To explore whether baseline brain structure is correlated with clinical phenotypes, voxel-based morphometry (VBM) will be employed to analyze the correlation between baseline gray matter volume and primary and secondary observed outcomes across all participants on a whole-brain scale. Age, sex, years of education, and total intracranial volume will be included as covariates in the regression model for control. Multiple comparison correction will be performed at the whole-brain voxel level using family-wise error rate (FWE), with a voxel-level P_FWE_ < 0.05 considered statistically significant.
2. Based on the brain regions showing significant correlations in analysis (1), gray matter volume in these regions was extracted for each participant to construct regions of interest (ROIs). A two-sample *t*-test was used to compare differences in gray matter volume between the two groups within the aforementioned ROIs, with age, gender, years of education, and total intracranial volume included as covariates. The Bonferroni method was used for multiple comparison correction.

#### 2.8.4. Correlation analysis

Spearman correlation analysis will be employed to explore the correlations between gray matter volume, gut microbiota, TMAO levels, and primary and secondary outcomes, respectively, in order to preliminarily reveal the potential mechanisms underlying the infrared moxibustion intervention effects. Bonferroni correction will be applied to adjust for multiple comparisons.

## 3. Discussion

Hyperlipidemia is a risk factor for cardiovascular disease and CD. Early management of HLP is crucial for preventing vascular complications and improving long-term outcomes. This randomized controlled trial aims to evaluate the therapeutic effect of infrared moxibustion on HLP and explore its mechanism of action through the gut microbiota-gut-brain axis.

The selection of mild hyperlipidemia patients for this study was driven by two primary considerations. First, hyperlipidemia is globally prevalent and serves as a risk factor for multiple diseases, including atherosclerosis [38], cognitive impairment [39], diabetes [40], and coronary heart disease [41], making its early prevention critically important. Patients with mild hyperlipidemia exhibit relatively mild lipid abnormalities, with metabolic disorders often in a reversible stage and fewer comorbidities with other severe metabolic diseases. This makes attempting reversal more feasible than in patients with severe complications. Second, there are currently no recommended medications for mild hyperlipidemia [36], with primary interventions being dietary control and lifestyle modifications. However, due to limited public awareness of food classification and low patient compliance and initiative in real-world clinical settings, complementary and alternative therapies requiring minimal patient involvement are highly necessary. If proven effective, this could provide a standardized, non-pharmaceutical complementary therapy option and establish preliminary evidence for exploring adjunctive therapeutic roles in moderate-to-severe patients.

The gut microbiota composition of patients with HLP differs significantly from that of healthy individuals [42]. Previous studies have demonstrated that moxibustion can modulate gut microbiota diversity in animal models or patients with hyperlipidemia, downregulating the abundance of TMA-producing bacterial groups (such as *Lachnospiraceae_NK4A136*, *Firmicutes*), and upregulating TMA-degrading bacteria (such as *Akkermansia*, *Enterococcus*, and *un_f_Muribaculaceae*) [34,43,44]. Gut microbiota-derived TMAO and its precursors are key metabolites mediating dyslipidemia [27,45], changes in brain volume [30], and cognitive impairmen [31]. Animal studies have further validated their role through fecal microbiota transplantation [46,47] and TMAO inhibitors [48,49]. An animal study showed that compared to normally fed rats, long-term high-fat diet-fed rats exhibit elevated blood lipids, impaired learning and memory, increased plasma and hippocampal TMAO levels, along with reduced glucose uptake capacity and volume in hippocampal tissue [50]. In clinical studies, serum triglyceride levels significantly differ between patients with cognitive impairment and those with normal cognition, with this difference significantly associated with reduced entorhinal cortex and hippocampal volumes [51]. A cross-sectional study by Chen Y et al. demonstrated a close correlation between TMAO, its precursor choline, and white matter hyperintensity volume [32]. Further a clinical cohort study found that plasma choline, a TMAO precursor, is significantly associated with reduced cognitive function, decreased total brain volume, and increased white matter hyperintensity volume, while carnitine is associated with reduced white matter hyperintensity volume [30]. Research by Gordon, S., et al. also suggests that TMAO levels in Puerto Rican adults may have a negative correlation with brain aging and hippocampal volume [52].

Regarding intervention studies, existing research indicates that traditional moxibustion can downregulate TMAO levels and improve lipid profiles in rabbits with atherosclerosis induced by a high-choline diet [33]. Lihong Fan et al. found significant differences in TMAO precursors (betaine and choline) between traditional moxibustion and model groups in hyperlipidemic rabbits, with significant correlations to Akkermansia [34]. Notably, Xiaojie Luo observed that after four weeks of acupuncture treatment, adolescent obese patients showed reduced gray matter volume in the superior frontal gyrus and cerebellar posterior lobe, increased gray matter volume in the right precentral gyrus, reduced white matter volume in the parahippocampal gyrus, pons, and precentral gyrus, and increased white matter volume in the right precuneus [35]. Other studies have found that acupuncture can induce changes in brain functional activity in obese patients and high-fat diet-induced obese rats [35,53,54]. Although obesity is often associated with dyslipidemia [55,56] and acupuncture can improve blood lipid levels in obese patients [57–60], it remains unclear whether acupuncture improves blood lipid levels in HLP patients by affecting brain structure. Based on this, this study explores the potential mechanisms by which infrared moxibustion improves blood lipids from multiple perspectives, including regulating gut microbiota abundance, affecting TMAO levels, and altering brain volume, to provide references for subsequent research.

Based on existing preclinical and clinical evidence, we propose the following anticipated results. First, regarding the primary outcome, we anticipate that an 8-week IM intervention will significantly reduce LDL-C levels in patients with mild HLP compared to the sham control group. This expectation is similar with previous clinical studies. Second, concerning secondary outcomes, we expect that IM will simultaneously improve cognition, emotional state, and sleep quality in participants, consistent with previous research findings [61–63]. Patients with hyperlipidemia often suffer from impaired vascular endothelial function and increased blood viscosity, leading to cerebral microcirculatory disturbances. Previous studies have demonstrated that infrared moxibustion can promote blood circulation and reduce blood viscosity [64], which may, to a certain extent, enhance cognitive function, alleviate anxiety and depression, and improve sleep quality in patients. Finally, regarding exploratory mechanistic outcomes, we anticipate that the mechanisms underlying the efficacy of infrared moxibustion may involve the regulation of gut microbiota structure, reduction of the gut microbiota metabolite TMAO levels, and improvement in brain volume.

The findings of this study hold significant clinical translational value and public health implications. On the one hand, IM is expected to serve as a complementary therapy for clinical intervention in mild hyperlipidemia, effectively delaying the progression of dyslipidemia, reducing the long-term risk of cardiovascular and cerebrovascular diseases as well as cognitive impairment, and alleviating the public health and medical burden. On the other hand, IM devices are cost-effective and easy to operate. following standardized training, they can be widely adopted in community health centers and even for home use. This is particularly applicable in regions with uneven distribution of medical resources, demonstrating strong grassroots applicability and public health value.

However, this study has several limitations. Firstly, the inclusion of various types of hyperlipidemias, including hypercholesterolemia, hypertriglyceridemia, and mixed hyperlipidemia, results in a relatively complex distribution of disease types. Nevertheless, this study can preliminarily establish the efficacy of infrared moxibustion for HLP. Future research can build upon these findings to focus on the effects of infrared moxibustion on different types of HLP. Secondly, the sample size of this study was calculated based on LDL-C, and its power for whole-brain neuroimaging analysis was insufficient. Therefore, it is necessary to conduct large-sample studies in the future for further verification. Furthermore, the association between gut microbiota and lipid metabolism is time-dependent and complex. Short-term interventions may lead to asynchronous changes between the two. The correlation analysis in this study can only preliminarily verify their association. Future studies with longer intervention periods and follow-up durations are needed to clarify the long-term causal relationship between them.

## Supplementary Material

SPIRIT.doc

Revised Manuscript_clean copy_269439281.docx

## Data Availability

The data can be requested from the corresponding author after formal publication of this trial.

## References

[1] Zhou Z, Moran C, Murray AM, et al. Association of year-to-year lipid variability with risk of cognitive decline and dementia in community-dwelling older adults. Neurology. 2025;104(4):e210247. doi: 10.1212/WNL.0000000000210247. 39879572 PMC11774555

[2] Ferhatbegović L, Mršić D, Kušljugić S, et al. LDL-C: the only causal risk factor for ASCVD. why is it still overlooked and underestimated? Curr Atheroscler Rep. 2022;24(8):635–642. doi: 10.1007/s11883-022-01037-3. 35635632

[3] Tynkkynen J, Chouraki V, van der Lee SJ, et al. Association of branched-chain amino acids and other circulating metabolites with risk of incident dementia and Alzheimer’s disease: a prospective study in eight cohorts. Alzheimers Dement. 2018;14(6):723–733. doi: 10.1016/j.jalz.2018.01.003. 29519576 PMC6082422

[4] Proitsi P, Kim M, Whiley L, et al. Association of blood lipids with Alzheimer’s disease: a comprehensive lipidomics analysis. Alzheimers Dement. 2017;13(2):140–151. doi: 10.1016/j.jalz.2016.08.003. 27693183

[5] Broce IJ, Tan CH, Fan CC, et al. Dissecting the genetic relationship between cardiovascular risk factors and Alzheimer’s disease. Acta Neuropathol. 2019;137(2):209–226. doi: 10.1007/s00401-018-1928-6. 30413934 PMC6358498

[6] Ma YH, Shen XN, Xu W, et al. A panel of blood lipids associated with cognitive performance, brain atrophy, and Alzheimer’s diagnosis: a longitudinal study of elders without dementia. Alzheimers Dement. 2020;12:e12041. 10.1002/dad2.12041 PMC7507431 32995461

[7] Dang L, Wei S, Zhao Y, et al. Effects of probucol on plasma amyloid-β transport in patients with hyperlipidemia: a 12-week randomized, double-blind, placebo-controlled trial. Lipids Health Dis. 2024;23(1):410. doi: 10.1186/s12944-024-02398-1. 39702132 PMC11657980

[8] Zong P, Li C, Feng J, et al. TRPM2 overactivation drives hyperlipidemia-induced dysfunction of myeloid cells and neurovascular units. Cell Rep Med. 2025;6(3):101998. doi: 10.1016/j.xcrm.2025.101998. 40056905 PMC11970404

[9] Wang Z, Ge Q, Wu Y, et al. Impairment of long-term memory by a short-term high-fat diet via hippocampal oxidative stress and alterations in synaptic plasticity. Neuroscience. 2020;424:24–33. doi: 10.1016/j.neuroscience.2019.10.050. 31711814

[10] Lord J, Green R, Choi SW, et al. Disentangling independent and mediated causal relationships between blood metabolites, cognitive factors, and Alzheimer’s disease. Biol Psychiatry Glob Open Sci. 2022;2(2):167–179. doi: 10.1016/j.bpsgos.2021.07.010. 36325159 PMC9616368

[11] Armstrong NM, An Y, Beason-Held L, et al. Predictors of neurodegeneration differ between cognitively normal and subsequently impaired older adults. Neurobiol Aging. 2019;75:178–186. doi: 10.1016/j.neurobiolaging.2018.10.024. 30580127 PMC6394867

[12] Wang H, Eckel RH. What are lipoproteins doing in the brain? Trends Endocrinol Metab. 2014;25(1):8–14. doi: 10.1016/j.tem.2013.10.003. 24189266 PMC4062975

[13] Wang Q, Zang F, He C, et al. Dyslipidemia induced large-scale network connectivity abnormality facilitates cognitive decline in the Alzheimer’s disease. J Transl Med. 2022;20(1):567. doi: 10.1186/s12967-022-03786-w. 36474263 PMC9724298

[14] Lipnicki DM, Sachdev PS, Crawford J, et al. Risk factors for late-life cognitive decline and variation with age and sex in the Sydney memory and ageing study. PLOS One. 2013;8(6):e65841. doi: 10.1371/journal.pone.0065841. 23799051 PMC3683032

[15] Kazibwe R, Rikhi R, Mirzai S, et al. Do statins affect cognitive health? A narrative review and critical analysis of the evidence. Curr Atheroscler Rep. 2024;27(1):2. doi: 10.1007/s11883-024-01255-x. 39520593 PMC11550230

[16] Korsholm MB, Pødenphanth TW, Strømgaard SK, et al. Are statins making older persons weaker? A discontinuation study of muscular side effects. Geroscience. 2024;46(1):853–865. doi: 10.1007/s11357-023-00817-2. 37225942 PMC10828417

[17] Vinci P, Di Girolamo FG, Pellicori F, et al. Statin-intolerant patients exhibit diminished muscle strength regardless of lipid-lowering therapy. J Clin Med. 2025;14(4):1221. doi: 10.3390/jcm14041221. 40004752 PMC11856913

[18] Mugawar B, McErlean S, P OC, et al. Statin intolerance and the drucebo effect. QJM. 2025;118(3):143–145. doi: 10.1093/qjmed/hcae144. 39067041

[19] Jareebi MA, Humedi A, Darraj A, et al. Evaluating the effectiveness of moxibustion in hyperlipidemia: a systematic review and meta-analysis. Med Princ Pract. 2026;35(2):126–143. doi: 10.1159/000548187. 40875704 PMC12503886

[20] Qian L, Han P, Honghua L, et al. Hypercholesterolemia treated with medicinal pad-separated moxibustion: a randomized clinical trial. World J Acupuncture – Moxibustion. 2022;32:310–316.

[21] Shao Q, Cheng J, Li Y, et al. Liquid chromatography-mass spectrometry-based plasma metabolomics study of the effects of moxibustion with seed-sized moxa cone on hyperlipidemia. Evid Based Complement Alternat Med. 2020;2020(1):1231357. doi: 10.1155/2020/1231357. 32047520 PMC7001670

[22] Proctor C, Thiennimitr P, Chattipakorn N, et al. Diet, gut microbiota and cognition. Metab Brain Dis. 2017;32(1):1–17. doi: 10.1007/s11011-016-9917-8. 27709426

[23] Xia M, Xu Y, Li H, et al. Structural and functional alteration of the gut microbiota in elderly patients with hyperlipidemia. Front Cell Infect Microbiol. 2024;14:1333145. doi: 10.3389/fcimb.2024.1333145. 38812752 PMC11133514

[24] Shanmugham M, Bellanger S, Leo CH. Gut-derived metabolite, trimethylamine-N-oxide (TMAO) in cardio-metabolic diseases: detection, mechanism, and potential therapeutics. Pharmaceuticals. 2023;16(4):504. doi: 10.3390/ph16040504. 37111261 PMC10142468

[25] Szudzik M, Zajdel M, Samborowska E, et al. High-fat diet with normal caloric intake elevates TMA and TMAO production and reduces microbial diversity in rats. Nutrients. 2025;17(13):2230. doi: 10.3390/nu17132230. 40647334 PMC12252406

[26] Yoo W, Zieba JK, Foegeding NJ, et al. High-fat diet-induced colonocyte dysfunction escalates microbiota-derived trimethylamine N-oxide. Science. 2021;373(6556):813–818. doi: 10.1126/science.aba3683. 34385401 PMC8506909

[27] Fei’erdun T, Zhang W, Yilihamujiang K, et al. Correlation between plasma trimethylamine N-oxide and lipid levels in hyperlipidemic patients. Sichuan Da Xue Xue Bao Yi Xue Ban. 2023;54:1030–1034. 37866964 10.12182/20230960109 PMC10579080

[28] Xiaoxing M. The role and mechanism of gut microbiota and its metabolites in long term high-fat diet-induced AD pathologies. Wuhan: Huazhong University of Science and Technology; 2023.

[29] Xie H, Jiang J, Cao S, et al. The role of gut microbiota-derived trimethylamine N-oxide in the pathogenesis and treatment of mild cognitive impairment. Int J Mol Sci. 2025;26(3):1373. doi: 10.3390/ijms26031373. 39941141 PMC11818489

[30] Yaqub A, Vojinovic D, Vernooij MW, et al. Plasma trimethylamine N-oxide (TMAO): associations with cognition, neuroimaging, and dementia. Alzheimers Res Ther. 2024;16(1):113. doi: 10.1186/s13195-024-01480-1. 38769578 PMC11103865

[31] Long C, Li Z, Feng H, et al. Association of trimethylamine oxide and its precursors with cognitive impairment: a systematic review and meta-analysis. Front Aging Neurosci. 2024;16:1519363. doi: 10.3389/fnagi.2024.1465457. 39629476 PMC11613963

[32] Chen Y, Xu J, Pan Y, et al. Association of trimethylamine N-oxide and its precursor with cerebral small vessel imaging markers. Front Neurol. 2021;12:648702. doi: 10.3389/fneur.2021.648702. 33868152 PMC8047127

[33] Bingbing Z. Exploring the effect of herbal-cake moxibustion on intestinal flora in atherosclerotic rabbits based on TMAO [Master thesis]. Hunan: Hunan University of Chinese Medicine; 2020.

[34] Lihong F, He H, Fang F, et al. Study on the mechanism of herbal cake-separated moxibustion in treatment of hyperlipidemia based on intestinal flora and metabonomics. China J Trad Chin Med Pharmacy. 2025;40:3816–3821.

[35] Xiaojie L. Neural mechanism study for obese adolescence: evaluated with MR-neuroimages and laboratory animals [Doctor]. China: Peking Union Medical College; 2013.

[36] Miao T, Zhang X, Zhang C, et al. Type 3 resistant starch from *Canna edulis* reduce lipid levels in patients with mild hyperlipidemia through altering gut microbiome: a double- blind randomized controlled trial. Pharmacol Res. 2024;205:107232. doi: 10.1016/j.phrs.2024.107232. 38825157

[37] Mi S, Xiaoming Z, Qiwen T. Intervention study of moxibustion combined with health belief mode on hyperlipidemia of turbid phlegm retention type. Shandong J Tradit Chin Med. 2022;41:759–763.

[38] Yang ST, Liu CH, Wang PH. The impact of hyperlipidemia and carotid atherosclerosis. J Chin Med Assoc. 2023;86(4):451–452. doi: 10.1097/JCMA.0000000000000909. 36823711 PMC12755513

[39] Sun R, Liu Y. The causal association between hyperlipidemia and Alzheimer disease: a combined NHANES and Mendelian randomization study. Medicine. 2025;104(43):e45393. doi: 10.1097/MD.0000000000045393. 41137217 PMC12558221

[40] Karmali R, Sipko J, Majid M, et al. Hyperlipidemia and cardiovascular disease in people with type 1 diabetes: review of current guidelines and evidence. Curr Cardiol Rep. 2023;25(5):435–442. doi: 10.1007/s11886-023-01866-x. 37052761

[41] Zeng Y, Zhao J, Zhang J, et al. Development of a nomogram that predicts the risk of coronary heart disease in patients with hyperlipidemia. J Cardiovasc Pharmacol Ther. 2023;28:10742484231167754. doi: 10.1177/10742484231167754. 37097005

[42] Hanchen D, Yifan X, DI C, et al. Analysis of gut microbiota composition changes in dyslipidemic populations under conventional dietary conditions. Food Nutr China. 2026;32:191–197 + 41.

[43] Xinyu C, Hongfen Y, Han P, et al. Exploring the effects of herbal cake-insulated moxibustion on gut microbiota in atherosclerotic rabbits based on 16S rRNA sequencing. Shanghai J Acupuncture Moxibustion. 2026;45:77–85.

[44] Huawen W, Ying Z, Min C. Effect of Jianpi Qutan recipe combined with moxibustion therapy on RCT metabolic pathway and gut microbiota in patients with hyperlipidemia. Liaoning J Tradit Chin Med. 2024;51:150–155 + 222.

[45] Tan CX, Chong GH, Hamzah H, et al. Effect of virgin avocado oil on diet-induced hypercholesterolemia in rats via (1) H NMR-based metabolomics approach. Phytother Res. 2018;32(11):2264–2274. doi: 10.1002/ptr.6164. 30051518

[46] Huang F, Zhang F, Xu D, et al. *Enterococcus faecium* WEFA23 from infants lessens high-fat-diet-induced hyperlipidemia via cholesterol 7-alpha-hydroxylase gene by altering the composition of gut microbiota in rats. J Dairy Sci. 2018;101(9):7757–7767. doi: 10.3168/jds.2017-13713. 29935822

[47] Zheng X, Zhang Z, Shan T, et al. Study on the mechanism of *Bifidobacterium animalis* subsp. lactis F1-3-2 regulating bile acid metabolism through TMA-TMAO pathway to improve atherosclerosis. Probiotics Antimicrob Proteins. 2025;17(6):4851–4866. doi: 10.1007/s12602-024-10417-x. 39708191

[48] Qi W, Xue L, Huihui L, et al. Exploration of the effect of intestinal-derived metabolite TMAO on brain neurotransmitter receptor gene changes in splenic-deficient hyperlipidemic rats under the perspective of brain-gut interaction based on PCR-microarray technique. J Liaoning Univ Tradit Chin Med. 2025;27:1–5.

[49] Xue L, Yang L. Effect of TMAO on lipid metabolism in spleen-deficiency hyperlipemia rats and the therapeutic effect of Xiangsha Liujunzi Decoction. Acta Laboratorium Animalis Scientia Sinica. 2023;31:1261–1270.

[50] Xiaoxing M. the role and mechanism of gut microbiota and its metabolites in long term high-fat diet-induced AD pathologies [Doctor]. Huazhong: Huazhong University of Science and Technology Doctoral Dissertation; 2023.

[51] Bernath MM, Bhattacharyya S, Nho K, et al. Serum triglycerides in Alzheimer disease: relation to neuroimaging and CSF biomarkers. Neurology. 2020;94(20):e2088–e98. doi: 10.1212/WNL.0000000000009436. 32358220 PMC7526673

[52] Gordon S, Lee JS, Scott TM, et al. Metabolites and MRI-derived markers of dementia risk in a Puerto Rican cohort. Metabolomics. 2025;21:161. doi: 10.1007/s11306-025-02240-4. 41233603

[53] He Y, von Deneen KM, Li G, et al. Electroacupuncture enhances resting-state functional connectivity between dorsal caudate and precuneus and decreases associated leptin levels in overweight/obese subjects. Brain Imaging Behav. 2022;16(1):445–454. doi: 10.1007/s11682-021-00519-3. 34415492

[54] Ren Y, Xu M, von Deneen KM, et al. Acute and long-term effects of electroacupuncture alter frontal and insular cortex activity and functional connectivity during resting state. Psychiatry Res Neuroimaging. 2020;298:111047. doi: 10.1016/j.pscychresns.2020.111047. 32114310

[55] Nussbaumerova B, Rosolova H. Obesity and dyslipidemia. Curr Atheroscler Rep. 2023;25(12):947–955. doi: 10.1007/s11883-023-01167-2. 37979064

[56] Zhu J, Zhang Y, Wu Y, et al. Obesity and dyslipidemia in Chinese adults: a cross-sectional study in Shanghai, China. Nutrients. 2022;14(11):2321. doi: 10.3390/nu14112321. 35684121 PMC9183153

[57] Huang D, Liu Z, Xu B, et al. Effect of acupuncture and moxibustion on severe obesity complicated with hyperlipidemia in different genders. Zhongguo Zhen Jiu. 2018;38(7):685–689. doi: 10.13703/j.0255-2930.2018.07.001. 30014659

[58] Yuan M, Liu Z, Xu B, et al. Effects of acupuncture on 1528 patients with obesity complicated with hyperlipidemia in different obesity levels. Zhongguo Zhen Jiu. 2016;36(8):807–811. doi: 10.13703/j.0255-2930.2016.08.008. 29231564

[59] Yan M, Zhu M, Quan F, et al. Acupoint thread-embedding at fat layer for abdominal obesity: a randomized controlled trial. Zhongguo Zhen Jiu. 2024;44(12):1370–1376. doi: 10.13703/j.0255-2930.20240517-k0005. 39658373

[60] Chen J, Gu Y, Yin L, et al. Network meta-analysis of curative efficacy of different acupuncture methods on obesity combined with insulin resistance. Front Endocrinol (Lausanne). 2022;13:968481. doi: 10.3389/fendo.2022.968481. 36120465 PMC9481269

[61] Mingyun M. Clinical research on effect of mild moxi-bustion on microcirculation in the patient of hyperlipidemia [Master thesis]. Nanjing: Nanjing University of Chinese Medicine; 2013.

[62] Xiaolu W. Clinical observation on the therapeutic effect of blood-letting cupping therapy combined with western medicine in treating hyperlipidemia of phlegm-obstruction type [Master thesis]. Shanghai: Shanghai University of Traditional Chinese Medicine; 2020.

[63] Jiankun Y, Guo W, Wenjin Y. Therapeutic observation of acupuncture-moxibustion for different levels of obesity coupled with hyperlipidemia. Shanghai J Acupuncture Moxibustion. 2019;38:1109–1113.

[64] Xia L, Chi Z, Honghua L, et al. Effect of whole body moxibustion on hemorheology in healthy adult. Chin Med Modern Distance Educ China. 2018;16:120–122.

